# Rhein Elicits *In Vitro* Cytotoxicity in Primary Human Liver HL-7702 Cells by Inducing Apoptosis through Mitochondria-Mediated Pathway

**DOI:** 10.1155/2015/329831

**Published:** 2015-06-14

**Authors:** Guy-Armel Bounda, Wang Zhou, Dan-dan Wang, Feng Yu

**Affiliations:** ^1^Department of Clinical Pharmacy, China Pharmaceutical University, No. 24, Tong Jia Xiang, Jiangsu, Nanjing 210009, China; ^2^Department of Pharmacology, China Pharmaceutical University, No. 24, Tong Jia Xiang, Jiangsu, Nanjing 210009, China; ^3^Key Laboratory of Drug Quality Control and Pharmacovigilance, China Pharmaceutical University, Ministry of Education, Nanjing 210009, China

## Abstract

*Objective*. To study rhein-induced apoptosis signaling pathway and to investigate its molecular mechanisms in primary human hepatic cells. *Results*. Cell viability of HL-7702 cells treated with rhein showed significant decrease in dose-dependent manner. Following rhein treatment (25 *μ*M, 50 *μ*M, and 100 *μ*M) for 12 h, the detection of apoptotic cells was significantly analyzed by flow cytometry and nuclear morphological changes by Hoechst 33258, respectively. Fatty degeneration studies showed upregulation level of the relevant hepatic markers (*P* < 0.01). Caspase activities expressed significant upregulation of caspase-3, caspase-9, and caspase-8. Moreover, apoptotic cells by rhein were significantly inhibited by Z-LEHD-FMK and Z-DEVD-FMK, caspase-9 inhibitor, and caspase-3 inhibitor, respectively. Overproduction of reactive oxygen species, lipid peroxidation, and loss of mitochondrial membrane potential were detected by fluorometry. Additionally, NAC, a ROS scavenger, significantly attenuated rhein-induced oxidative damage in HL-7702 cells. Furthermore, real-time qPCR results showed significant upregulation of p53, PUMA, Apaf-1, and Casp-9 and Casp-3 mRNA, with no significant changes of Fas and Cytochrome-c. Immunoblotting revealed significant Cytochrome-c release from mitochondria into cytosol and no change in Fas expression. *Conclusion*. Taken together, these observations suggested that rhein could induce apoptosis in HL-7702 cells via mitochondria-mediated signal pathway with involvement of oxidative stress mechanism.

## 1. Introduction

*Polygonum multiflorum *Thunb. (PMT, Polygonaceae) also known as Fo-Ti is a traditional Chinese herbal medicine common in northeast Asia. Its roots have been widely used as therapeutic agent including antiallergy, antitumor, antibacterial, spasmolytic, antialopecia, vasorelaxant, and anti-aging agent for many centuries in Asian traditional medicine [1–4]. Mounting data of pharmacological effects of this herb and its components, including anti-inflammation, antioxidative, and neuroprotective, as well as improved learning and memory, have been recently published [5–8]. The genus* Polygonum *is the source of a wide range of phenolic compounds, flavonoids, anthraquinones, stilbenes, and tannins [9], including a number of anthraquinones in the stilbene class such as (*E*)-2,3,4′,5-tetrahydroxystilbene-2-*β*-D-glucoside, rhein, emodin, aloe-emodin, chrysophanol, physcion, and their derivatives [10].

In recent years, drug-induced liver injury (DILI) has been one of the interesting topics which have led to conducting several researches on herbal medicines. Published data have reported the toxicological effect of* Polygonum multiflorum* on the liver [11, 12]. In liver, like other organs, apoptosis plays a key role during physiological cell renewal [13, 14] and in cellular depletion after stimulation with mitogens or hyperplasia-inducing treatments [15]. A growing number of published evidences suggest that hepatocyte apoptosis can contribute to the development of many liver diseases, including alcoholic liver injury, chronic viral hepatitis, cholestatic liver diseases, and hepatic fibrosis [8, 16, 17].

With thousands of years of medical practice, Traditional Chinese Medicine (TCM) has accumulated rich theories (including yin yang, ch'i (qi), meridian, five-phase (or five-element), and zang-fu theories) and a great deal of valuable experience in the prevention and treatment of several diseases or medical condition [18, 19]. According to the theory of TCM, the liver is regarded as a special organ in the body; it is related to the eyes via the meridian connections, and its condition is reflected in the nails. It is involved by virtue of its role in regulating or ensuring the free flow of qi around the body and its role in regulating blood volume (“storing blood”) [20]. Based on long period of traditional and clinical practice, TCM considered that the rhizome of* Polygonum multiflorum* possesses sweet and slightly warm properties, making it especially suitable for treating patients who have not only blood deficiency but also a mild Yang and Qi deficiency of the body. It opens the meridians and collaterals; able to tonify the blood as well the kidney essence (Jing), strengthens the tendons and bones, and improves sleep [21]. TCM theory said that both graying of hair and hair loss may be due to lack of essential essences, explaining why* Polygonum multiflorum* has been used to promote the growth of hair and to treat premature greying of hair, which is done by nourishing the kidney's “yin” energy, replenishing and cooling down the blood [22]. However, the long-term use of* Polygonum multiflorum* may lead to liver and kidney toxicity as several clinical cases of hepatotoxicity have been linked to its consumption.

**Figure 1:**
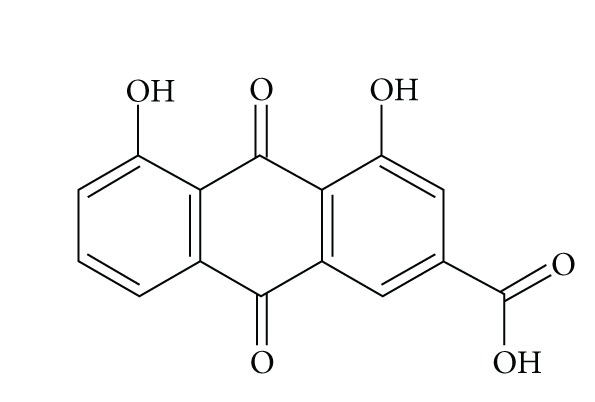
Chemical structure of rhein (4, 5-dihydroxyanthraquinone-2-carboxylic acid). Molecular formula: C_15_H_8_O_6_. Molecular weight: 284.22 g/mol.

Rhein (4, 5-dihydroxyanthraquinone-2-carboxylic acid, Figure 1) is one of the most important bioactive components of PMT. Mounting published reports demonstrated that its pharmacological effects including anti-inflammatory [8], antiallergic [23], antifungal [24], antibacterial [25], antiviral [26], and anticancer ones [27–29]. Recently, the suppression of Hep-G2 cells proliferation induced by rhein was expressed* via* mitochondrial permeability transition, but the oxidative stress injury mechanism was not investigated in this study [30]. Mostly the mechanism of rhein antitumor activity in cancer cells listed previously is commonly due to its ability to induce apoptosis in corresponding cancer cells [28, 29].

Apoptosis or programmed cell death (PCD), a genetically controlled process whereby cells die in response to environmental or developmental cues, contributes to the pathogenesis of disease or removal of cells in adult organism [31]. It is characterized by the activation of biochemical pathways that lead to changes in cellular morphology, DNA fragmentation, perturbation of mitochondrial membrane function, decrease of mitochondrial membrane potential, translocation of phosphatidylserine (PS) to the external cell surface, and changes in the plasma membrane [32]. Understanding and regulation of apoptosis are critical for normal development and tissue homeostasis, and disruption of this process can have severe consequences [33]. Too much cell death may produce neurodegenerative diseases and impaired development, while insufficient cell death can lead to increased susceptibility to cancer and sustained viral infection [34]. Numerous scientific data have proven that apoptotic signaling within the cell may occur by two fundamental pathways: (1) death receptor or extrinsic pathway and (2) the mitochondria or intrinsic pathway [35]. Rhein has been investigated and shown to induce cytotoxicity and apoptosis in primary cultures of rat hepatocytes [36]. Measurement of apoptosis has become an essential component of the evaluation of cytotoxicity of chemicals [31].

Liver cells, especially hepatocytes, are notable for their wide variety of metabolic and other functional capacities, spanning over 500 classes of functions such as energy metabolism, bile production, and synthetic or detoxification functions [37]. Primary human hepatocytes remain differentiated and sustain the major drug-metabolizing enzymes activities; they represent a unique* in vitro* system and serve as a “gold standard” for studies of drug metabolism and toxicity [38]. HL-7702 cell expressed a distinct ultrastructure compared to hepatic carcinoma and is considered an ideal* in vitro* model of a primary Chinese nonmalignant liver [39].* In vitro* and* in vivo* studies have been conducted to assess the biosynthetic activities of HL-7702 cells in order to explore the possibility to use this cell line for a liver support system. In a 7-day* in vitro* study done by Yang et al. [40], the authors found that HL-7702 cells could keep their function of protein synthesis by culturing on thermoresponsive hydrogen. Albumin secretion continuously grew and the function of urea synthesis was significantly increased with in time delayed from (0.16 ± 0.02) *μ*mol/mL to (0.41 ± 0.04) *μ*mol/mL [40]. Furthermore, the proliferation state of cells by cell cycle analysis was proven to not be damaged. In another study, conducted by Hu and colleagues [41], the biosynthetic function of HL-7702 cells in terms of albumin, uridine diphosphate glucuronosyltransferase (UGT), and cytochrome P450 3A4 gene and protein was significantly expressed. In the same study, in an* in vivo* acute liver failure (ALF) model established by 90% partial hepatectomy, rats transplanted with HL-7702 cells showed significantly improved survival of 70% versus 0% in controls (*P* < 0.01). Moreover, the enzymatic analysis of various enzymes or liver markers such as albumin, alanine transaminase, aspartate transaminase, serum ammonia, alkaline phosphates, and total and direct bilirubin revealed a significant improvement compared to the control groups [41]. These evidences support that HL-7702 cells could proliferate and keep their biosynthetic functions at the same time, suggesting them to be a feasible source for liver support system and ideal for pharmacological and toxicological studies.

Despite few toxicological studies done on HL-7702 cells (also known as L-02 cells) and knowing the paradoxical hepatotoxicity and hepatoprotection of rhein, no available study had ever addressed the effects of rhein on apoptosis in primary human liver HL-7702 line cell. Here we show some evidence about the missing information using primary human hepatic cell line HL-7702 cells, which is one of the commonly used human primary liver cells for* in vitro* evaluation apoptosis induced by drugs, as this primary human liver cell line has been well proven and studied in numerous researchers published data [40–43] to analyze the drug-induced hepatotoxicity. Thus, the aim of this study was to investigate the* in vitro* cytotoxic activity of rhein in HL-7702 cells and to assess the possible relation between liver injury and cellular uptake of rhein and possible mechanisms involved.

## 2. Materials and Methods

### 2.1. Reagents and Antibodies

Rhein (4, 5-dihydroxyanthraquinone-2-carboxylic acid, purity > 99.99%) was purchased from the Chinese National Institute for Food and Drug Control and then dissolved in dimethyl sulfoxide (DMSO, Sigma-Aldrich, USA) to a concentration of 20 mg/mL. DMEM high glucose (Dulbecco's modified Eagle's medium, high glucose, containing L-glutamine), FBS (fetal bovine serum), Maxima SYBR Green qPCR Master Mix, and trypsin were obtained from Thermo Fischer Scientific, USA. 3-(4,5-Dimethylthiazol-2-yl)-2,5-di-phenyl tetrazolium bromide (MTT) and the total RNA extraction reagent were purchased from Nanjing Sunshine Biotechnology (China). Annexin V-FITC/PI apoptosis detection kit, alanine aminotransferase (ALT), and aspartate aminotransferase (AST) assay kits were obtained from Nanjing Jiancheng Bioengineering Institute. The triglyceride (TG) reagent assay and total cholesterol (TC) reagent assay kits were purchased from Zhejiang Dong'ou Diagnostic Products Co., Ltd. The cell lysis buffer for western and PI, phenylmethylsulfonyl fluoride (PMSF), LDH cytotoxicity assay kit, trypan blue, JC-1 mitochondrial membrane potential detection kit, Hoechst Staining Kit, enhanced BCA protein kit, cell mitochondria isolation kit, reactive oxygen species assay kit, N-acetyl-L-cysteine (NAC), lipid peroxidation (MDA) assay kit, total superoxide dismutase (SOD) assay kit, and caspase-3, -9, and -8 activity assays kits were purchased from Beyotime Institute of Biotechnology, China. The various caspase inhibitors, Z-IETD-FMK (caspase-8), Z-LEHD-FMK (caspase-9), and Z-DEVD-FMK (caspase-3) were purchased from Calbiochem-Novabiochem Co., (San Diego, CA, USA). AMV First Strand cDNA Synthesis Kit and all the primers were designed and synthesized by Shanghai Sangon Biotechnology Co., Ltd. The primary antibodies (including anti-Fas, anti-Cytochrome-c, and anti-*β*-actin) and secondary antibody conjugated to horseradish peroxidase were purchased from Cell Signaling Technology, Inc. USA. All other reagents were of analytical grade.

### 2.2. Cells Line and Cells Culture

Primary human liver HL-7702 cells, also known as L-02 cells, were purchased from the Institute of Biochemistry and Cell Biology Sciences, Chinese Academy of Sciences (Shanghai, China). Expressing a distinct ultrastructure compared to hepatic carcinoma cells, HL-7702 cells are considered an ideal* in vitro* model of Chinese nonmalignant liver cells [39, 44, 45]. These cells were routinely grown as monolayer in DMEM containing 4500 mg/L glucose, supplemented with 10% fetal bovine serum, and maintained at 37°C in a cell culture humidified incubator with 95% air and 5% CO_2_. The HL-7702 cells were used in all experiments described below here.

### 2.3. Determination of Cell Viability

MTT assay was used to assess cell viability as a function of redox potential, as only viable cells have functioning mitochondrial dehydrogenase enzymes which can reduce MTT to formazan [46]. Briefly, HL-7702 cells were seeded in 96-well microplates at a cell density of 8 × 10^3^ cells per well. After pretreatment with different concentrations of rhein for 24 and 48 h, respectively, cell viability was assessed by incubating cells with 20 *μ*L of MTT (5 mg/mL) for 4 h, at 37°C. The medium was then removed and replaced by 150 *μ*L of DMSO in each culture and mixed by pipetting, and the plates were then vibrated for 10 min to uniformly dissolve the crystals. Absorbance readings were performed at 570 nm using a microplate reader (Molecular Devices, USA) with the optical density (OD). The results were calculated with the following formula: survival rate (%) = (OD treated well − OD blank)/(OD control well − OD blank) × 100%.

### 2.4. Lactate Dehydrogenase (LDH) Assay

After treatment with different concentrations of rhein for 24 and 48 h, respectively, LDH activity was measured by using a LDH cytotoxic assay kit (Beyotime Institute of Biotechnology, China) according to the manufacturer's protocol. Briefly, the HL-7702 cells were seeded on 12-well cultures plates at a density of 1.5 × 10^6^ cells/well. At the end of the treatment, the medium was collected to assess the LDH activity. To determine the intracellular LDH activity, the cells were washed by PBS and then 150 *μ*L PBS were added into each well and the cells were lysed with 200 *μ*L of 0.1% Triton X-100. LDH activities in both the culture supernatants and the cell lysates were determined by adding 60 *μ*L of substrate solution from the kit, followed by incubation at 25°C for 30 min. The absorbance of the samples was recorded at 490 nm. The LDH leakage was expressed as the percentage (%) of the total LDH activity (LDH in the medium + LDH in the cell), according to the equation % LDH released = (LDH activity in the medium/total LDH activity) × 100.

### 2.5. Assessment of Liver Marker Enzymes

Serum ALT and AST were determined using the commercial kits purchased from Jiancheng Institute of Biotechnology (Nanjing, China). Briefly, HL-7702 cells were placed and seeded in 96- well plates. The following day, medium was refreshed and the cells were treated with rhein (10 *μ*M–400 *μ*M) for 12 hours of incubation. At the end of incubation period, the medium was discarded; wells were gently washed twice with 1 mL PBS. Cells were centrifuged (1000 g, 10 min), and the supernatant was discarded. Cells were homogenized on ice in the correspondent assay buffer. ALT and AST levels in the collected cell lysates were analyzed using the colorimetric assay kit according to the manufacturer's instructions.

### 2.6. Nuclear Staining with Hoechst 33258

Cell death was assessed based on nuclear morphological changes that were determined following nuclei staining with Hoechst Staining Kit. Briefly, HL-7702 cells were seeded on glass cover slips in 6-well plates. At 12-hour treatment with rhein (25 *μ*M, 50 *μ*M, and 100 *μ*M), plates were rinsed twice with PBS and then fixed with stationary liquid for 10 min at room temperature. The cells were then stained with Hoechst 33258 solution in the dark for 5 min at 37°C. After washing twice with PBS, the morphological features of apoptosis (nuclear shrinkage, chromatin condensation, intense fluorescence, and nuclear fragmentation) were monitored by fluorescence microscopy with inverted Leica microscope and a UV filter (Leica 2500; Leica Corporation, Germany). Apoptotic cells were identified as those whose nuclei exhibited brightly staining condensed chromatin, nuclear fragmentation, or apoptotic bodies, while those with uniformly stained nuclei were identified as healthy.

### 2.7. Annexin V/PI Analysis for Cell Apoptosis

The Annexin V/PI double staining assay was further used to determine the apoptotic cells, according to the manufacturer's protocol. In brief, HL-7702 cells were plated in a 6-well culture plate at a density of 4.0 × 10^5^ cells/well and treated with rhein (25 *μ*M, 50 *μ*M, and 100 *μ*M) for 12 hours. At the end of the treatment, they were digested with trypsin and harvested by centrifugation and then resuspended in 500 *μ*L buffer solution. Then Annexin V-FITC/PI staining solution (PI (5 *μ*L) and Annexin V (5 *μ*L)) was added. After incubation for 10 min at room temperature in the dark, then the stained cells were analyzed in FACScan flow cytometry analyzer (Becton Dickinson Biosciences, CA, USA). Excitation wave was at 488 nm and the emitted green fluorescence of Annexin (FL1) and red fluorescence of PI (FL2) were measured using 525 nm and 575 nm band pass filters, respectively. A total of at least 10 000 cells were analyzed per sample. The amount of healthy cells, early apoptosis, and late apoptosis/necrosis were determined as the percentage of Annexin V−/PI−, Annexin V+/PI−, or Annexin V+/PI+ cells, respectively, using CellQuest software.

### 2.8. Effects of Rhein on Enzymes Involved in Lipid Metabolism

#### 2.8.1. Total Cholesterol (TC) and Triglycerides (TG) Assays

Briefly, the cells were plated in a 96-well culture plate and treated with rhein (25 *μ*M, 50 *μ*M, and 100 *μ*M) for 12 h. Collect 10 × 10^6^ cells by centrifugation at 1000 ×g for 10 minutes. Discard the supernatant and resuspend in 1 mL of cold PBS containing 1% Triton X-100. Homogenize or sonicate the cell suspension 20x at one-second bursts. Centrifuge cell suspension at 10000 ×g for 10 minutes at 4°C. Carefully collect the supernatant and should be stored on ice for immediate use. Cell lysates must be further diluted before assaying (1 : 5 or greater). The TC and TG levels in the collected cell lysates were analyzed using the colorimetric assay kit according to the manufacturer's instructions.

#### 2.8.2. Assessment of HGM-CoAR, ACoAC, and GPAT Relative Transcripts

Hydroxymethylglutaryl coenzyme A reductase (HMG-CoAR) regulates several pathways within animal cells, as it is the rate-limiting enzyme in the biosynthesis of cholesterol and represents the sole major drug target for contemporary cholesterol-lowering drugs. Acetyl-CoA carboxylase (ACoAC) plays a critical role in the regulation of long-chain fatty acid synthesis. The initial and rate-limiting step of glycerolipid synthesis is the acylation of glycerol-3-phosphate (G3P) with long-chain fatty acyl-CoA to form lysophosphatidic acid (LPA). This reaction is catalyzed by glycerol-3-phosphate acyltransferase (GPAT). To assess the toxicological effect of Rhein on the lipid biosynthesis in HL-7702 cells, the transcripts of these three enzymes were analyzed. The assay procedure is described in Section 2.13.

### 2.9. Mitochondrial Membrane Potential (Δ*φ*_mit_) Assay

Loss of mitochondrial membrane potential (Δ*φ*_mit_) was assessed by fluorescence spectrophotometry (Shimadzu, Japan), using the mitochondria-specific lipophilic cationic fluorescent dye JC-1. In healthy cells with high mitochondrial Δ*φ*_mit_, JC-1 spontaneously aggregates and gives intense red fluorescence [47]. On the other hand, in apoptotic or unhealthy cells with low Δ*φ*_mit_, JC-1 remains in the monomeric form, which shows only green fluorescence. Briefly, to monitor mitochondrial membrane potential Δ*φ*_mit_, cells grown in 96-well polystyrene culture plates were treated with different concentrations of rhein (25 *μ*M, 50 *μ*M, and 100 *μ*M) for 12 hours. Then, JC-1 staining working solution (5 *μ*g/mL) was added to the culture and then incubated in the dark at 37°C for 20 min. Cells were then washed twice with ice-cold PBS and then qualitatively and quantitatively analyzed by fluorescence spectrophotometry. 5 *μ*L of 10 *μ*M of the protonophore carbonyl cyanide m-chlorophenylhydrazone (CCCP) was a positive control that could induce mitochondria membrane depolarization. Laser scanning confocal microscope (Zeiss), with the excitation wavelength of 488 nm, emission of green, and red fluorescence intensity wavelength of 545 nm, was used for detection. The ratio of red/green fluorescence was calculated and presented in arbitrary units. A decrease in this ratio indicates mitochondrial depolarization (i.e., loss of Δ*φ*_mit_).

### 2.10. Measurement of Intracellular Reactive Oxygen Species (ROS) Production

The production of intracellular reactive oxygen species (ROS) was monitored by fluorescence spectrophotometer (Shimadzu, Japan) after staining with 2′7′-dichlorofluorescin diacetate (DCFH-DA). Briefly, after treatment with rhein (25 *μ*M, 50 *μ*M, and 100 *μ*M) for 12 hours or pretreated with 5 mM NAC for 1 hour, HL-7702 cells (1 × 10^6^ cells/mL) were washed twice with PBS and then incubated with the 2′7′-dichlorofluorescin diacetate (DCFH-DA) working solution (10 *μ*M final concentration) for 20 min at 37°C in the dark. The intensity of the fluorescence signal was then detected dose dependently at 488 nm excitation and 525 nm emission. 90%, by using trypan blue. The results of ROS production are expressed as increase in fluorescence in respect of control.

### 2.11. Determination of Lipid Peroxidation (MDA) and Superoxide Dismutase (SOD)

The cell culture and rhein treatment were conducted in the same manner described above. After rhein exposure, the cells were harvested, washed twice with PBS, and lysed in cell lysis buffer, centrifuged at 14,000 ×g for 5 min at 4°C. The lysates were then collected and stored at −20°C until further use. The supernatants were used for measuring cellular MDA and SOD using the commercially available assay kits (Beyotime Institute of Biotechnology, China). The MDA level was calculated by evaluating the thiobarbituric acid reacting substances at a wavelength of 532 nm. The activity of SOD was determined by making use of the hypoxanthine and xanthine oxidase system [43]. One unit of SOD activity was defined as the amount of enzyme required to inhibit oxidation by 50% in a 1 mL reaction, in the absorbance at 450 nm. All the operations process were done according to the manufacturer's instructions and measured with a microplate reader (VersaMax, USA). The protein concentration of each treatment group was determined using the BCA protein assay kit (Beyotime, China). The results for MDA and SOD were defined as *μ*M/*μ*g protein and U/*μ*g protein, respectively.

### 2.12. Caspase-3, -8, and -9 Activity Assay

Caspase-3, -8, and -9 activities were measured by colorimetric assay using the cleavage of a colorless substrate specific for caspase-3 (Ac-DEVD-*ρ*NA), caspase-8 (Ac-IETD-*ρ*NA) or caspase-9 (Ac-LEHD-*ρ*NA) releasing the chromophore, *ρ*-nitroaniline (*ρ*NA). Assays were carried out according to the manufacturer's instructions. Briefly, cell lysates were prepared after their respective treatment. Assays were performed on 96-well microtitre plates by incubating 20 *μ*L cell lysate protein per sample in 70 *μ*L reaction buffer containing 10 *μ*L caspase-3, -8, or -9 substrates. Lysates were incubated at 37°C for 1 h. The release of *ρ*NA was quantitated spectrophotometrically by measuring absorbance at 405 nm using a microplate reader (Molecular Devices, USA) and enzyme activity was calculated with reference to standard curve of *ρ*NA concentration versus absorbance. The data were represented as the U/mgPro. Protein content was measured according to Bradford method. Assays were done in triplicate.

To elucidate the antiapoptotic effects of caspase inhibitors on the apoptosis induced by rhein, cells were preincubated with 25 *μ*M Z-IETD-FMK (caspase-8), Z-LEHD-FMK (caspase-9), or Z-DEVD-FMK (caspase-3) for 1 h. Following incubation with rhein for 12 h, apoptosis was determined by FACS analyses.

### 2.13. RNA Extraction and Quantitative RT-PCR Assay

Total cellular RNA was extracted from cells using the TRIzol method to guarantee an OD260/280 ratio in the range of 1.8–2.0. RNA was reverse transcribed into single-stranded cDNA by the Revert Aid First Strand cDNA Synthesis Kit. The synthesized cDNA with primer and SYBR Green Master Mix (Thermo Fischer Scientific, USA) was then amplified by quantitative real-time PCR. qRT-PCR were performed using forward and reverse primers shown in Table 1 and run on a Mastercycle ep realplex real-time PCR system (Eppendorf, Germany). Glyceraldehyde-3-phosphate dehydrogenase (GAPDH) was used as an internal control in parallel for each run. The cycle number at which the fluorescent signal crosses the detection threshold was denoted as the threshold cycle (C_t_). All samples were run in triplicate and underwent 40 amplification cycles according to the manufacturer protocol (95°C for 10 min followed by 40 cycles at 95°C for 15 s, 60°C for 30 s, and 72°C for 30 s). Relative amounts of target RNA were quantified by the 2^−ΔΔCt^ method and normalized to the corresponding GAPDH values. Specificity of real-time PCR products was determined by melting curve analysis.

### 2.14. Protein Extraction and Western Blotting Analysis of Fas and Cyt-c

To determine the expression of associated proteins, western blotting was performed. HL-7702 cells were harvested after rhein treated (25 *μ*M, 50 *μ*M, and 100 *μ*M) for 12 h. The total proteins were extracted using Lysis buffer (PMSF was added previously to a total concentration of 1 mM). Cytosolic (deprived of mitochondria protein) and mitochondrial proteins were extracted using cell mitochondrial isolation kit, according to the manufacturer's protocol. Protein concentration was determined using enhanced BCA protein assay kit. The extracted protein samples were mixed with 5x SDS loading buffer and boiled for 5 min. They were separated on SDS-polyacrylamide gel electrophoresis (SDS-PAGE) and transferred electrophoretically to a 0.45 *μ*m (for Fas) or 0.22 *μ*m (for Cyt-c) nitrocellulose (NC) membranes (BOSTER, China). The membranes were blocked with TBS-T (Tris 20 mM, pH 7.6, NaCl 150 mM, and 0.1% Tween-20) containing 5% bovine serum albumin (BSA) for 1 h at room temperature, and the membranes were incubated with dilute solution (1 : 1000 in 5% w/v BSA, 1x TBS, and 0.1% Tween) of primary antibodies including anti-Fas, anti-Cytochrome-c, and anti-*β*-actin overnight at 4°C. After washing four times with TBST for 8 min, the membranes were incubated with secondary antibody (1 : 10000 dilution) conjugated to horseradish peroxidase for 1 h at room temperature. The membranes were then washed four times for 8 min with TBST. Immune-reactive proteins were detected using ECL western blotting detection system (Millipore, Germany) and visualized with the ChemiDoc XRS+ system (Bio-Rad Laboratories, USA). Densitometric analysis of immunoblots was performed by using Quantity One software and *β*-actin was used for standardization.

### 2.15. Statistical Analysis

All experiments were performed at least in 3 independent* in vitro* experiments (unless stated otherwise); all results are presented as the mean ± standard deviation (S.D.) and were processed with SPSS 18.0 software (SPSS, Chicago, IL). Statistical significance was assessed using a two-way ANOVA followed by Tukey's* post hoc* test. The significance level was set at *P* < 0.05. Error bars denote SD, unless stated otherwise.

## 3. Results

### 3.1. Rhein-Induced Inhibition of HL-7702 Cells Viability

**Figure 2:**
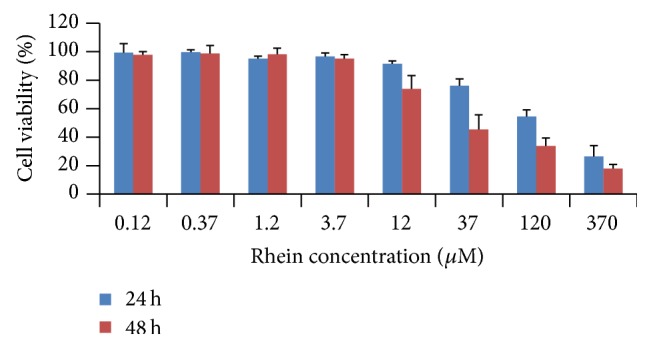
Rhein induces* in vitro* cytotoxicity of HL-7702 cells in a dose-and time-dependent manner. HL-7702 cells were incubated with different concentration of rhein for 24 h or 48 h and then processed for MTT assay. Cell viability was made relative to untreated control cells (100%). Data are expressed as mean ± SD from three independent experiments.

Cytotoxicity of rhein was assessed with the MTT assay. A dose and time-dependent relationship study of HL-7702 cells treated with a series of concentrations of rhein (0.12 *μ*M–370 *μ*M), respectively, for 24 h and 48 h was conducted. As shown in Figure 2, cell viability expressed both dose and time-dependent relationships. It significantly decreased after 24 h of rhein treatment with 37 *μ*M, 120 *μ*M, and 370 *μ*M concentrations. Meanwhile the cell viability greatly decreased after 48 h of rhein treatment with the following concentrations: 12 *μ*M, 37 *μ*M, 120 *μ*M, and 370 *μ*M. To compare the cell viability, IC_50_ values 131.79 *μ*M and 18.45 *μ*M were calculated, respectively, at 24 h and 48 h.

### 3.2. Rhein Damaged the Integrity of Cell Membrane

**Figure 3:**
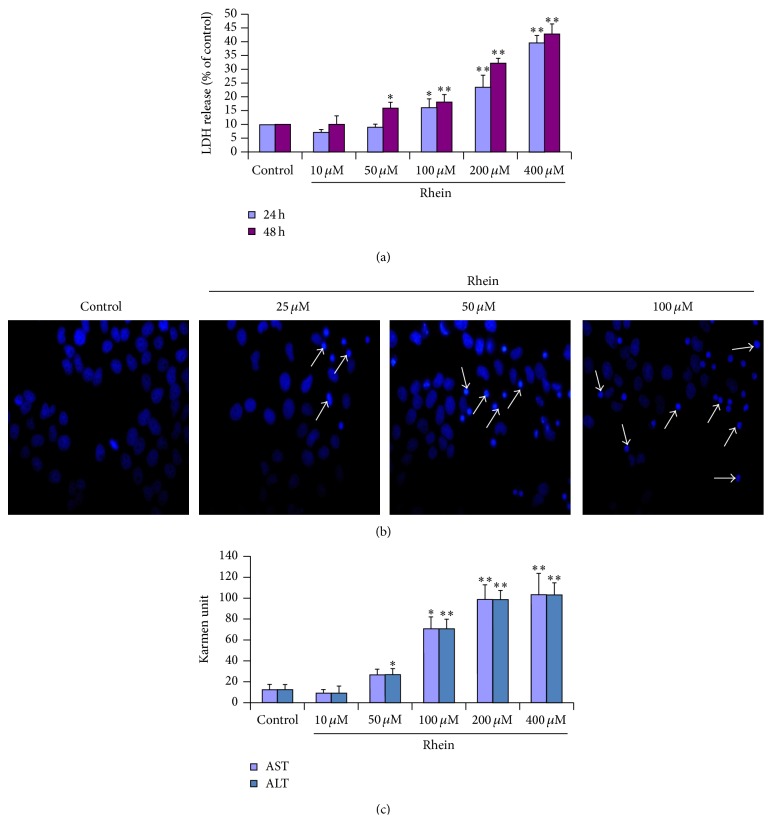
Rhein ravaged the integrity of cell membrane and induced cell morphological changes. (a) Lactate dehydrogenase assay was used to evaluate the extent of rhein cytotoxicity. The method was based in measuring LDH leakage in the culture medium after rhein treatment with various concentrations (10 *μ*M–400 *μ*M) during 24 h and 48 h. Values given are the mean ± SD from three independent experiments. (^*∗∗*^ *P* < 0.01 versus control and ^*∗*^ *P* < 0.05 versus control). (b) HL-7702 cells were stained with Hoechst 33258 and examined under fluorescent microscope (mag. 400x). Nuclei chromatin margination and condensation were shown by arrows. (c) HL-7702 cells were incubated with a wide range of doses of rhein for 12 h and then processed for AST and ALT colorimetric assay. Analysis of AST and ALT elevation was made relative to untreated control cells (100%). Data are expressed as mean ± SD from three independent experiments. (^*∗*^ *P* < 0.05 versus control and ^*∗∗*^ *P* < 0.01 versus control).

Lactate dehydrogenase (LDH) is abundant in the cytoplasm and cannot pass through the normal cell membrane but would be released into the extracellular medium if only cells are damaged or dead. Therefore, an elevated level of LDH leakage will reflect cell membrane damage. We treated cells with a series of rhein's concentrations (10 *μ*M–400 *μ*M), respectively, for 24 h and 48 h. The results are shown in Figure 3(a). These results showed that rhein was able to induce LDH leakage of HL-7702 cell in all groups in a dose- and time-dependent manner.

The cell membrane integrity was also further investigated using Hoechst 33258 fluorescent staining technique. The morphological characteristics of the human primary liver HL-7702 cells following treatment with rhein (25 *μ*M, 50 *μ*M, and 100 *μ*M) for 12 h were observed and then photographed under a fluorescence microscope. The results shown in Figure 3(b) confirmed that rhein ravaged the integrity of cell membrane, inducing apoptosis in HL-7702 cells in dose-dependent manner. The treated cells showed strong morphological alterations (nuclear shrinkage, chromatin condensation, and intense fluorescence). Condensed chromatin could also be found in many treated cells, which is one the classic characteristics of apoptotic cells.

ALT and AST are cytosolic enzymes in the liver, which serve as biomarkers of hepatocyte damage that are involved in various reactions in the liver. A considerable increase in the plasma levels of these enzymes indicates liver injury [44]. The liver markers enzymes assessment showed that ALT and AST activities increased considerably after 12 hours of treatment with rhein (50 *μ*M–400 *μ*M) (Figure 3(c)), indicating liver toxicity.

All these observations, to a certain extent, confirmed rhein exerted liver damage* in vitro* through ravaged integrity of cell membrane, thus rhein-induced apoptosis in HL-7702 cells.

### 3.3. Rhein-Induced Apoptosis in Primary Human HL-7702 Cells

**Figure 4:**
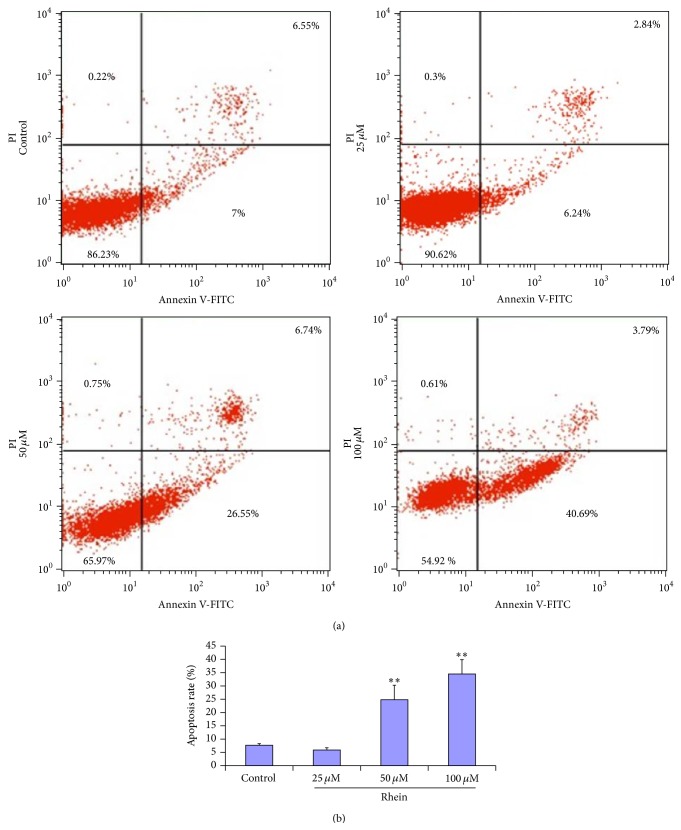
Apoptosis induced by rhein. (a) A representative result of flow cytometry of HL-7702 cells stained with Annexin V/PI, after treatment with rhein at concentration of 25 *μ*M, 50 *μ*M, and 100 *μ*M for 12 h (Annexin V−/PI− represents viable cells, Annexin V+/PI− represents early apoptotic cells, and Annexin V+/PI+ represents late apoptosis or necrotic cells). (b) The experiment was repeated three times and the percentage of early apoptotic cells (mean ± SD) for each treatment group is shown (^*∗∗*^ *P* < 0.01 versus control).

To further investigate and quantify the extent of apoptosis in the total cell population, the flow cytometric measurement was applied. Following the incubation of HL-7702 cells with three different concentrations (25 *μ*M, 50 *μ*M, and 100 *μ*M) of rhein for 12 h, the percentage of early apoptotic, late apoptotic/necrotic cells was assessed. Significant differences were observed between the control and the rhein-treated cells (Figure 4(b)). The percentage of early apoptotic cells in 25 *μ*M rhein-treated did not express a statistical significance when compared to the control group. But we observed a statistically significant increase in 50 *μ*M rhein-treated cells and in 100 *μ*M rhein-treated cells (Figures 4(a) and 4(b)). Taken together, these results demonstrated that rhein induced a dose-dependent apoptosis in HL-7702 cells.

### 3.4. Rhein Altered the Regulation of Enzymes Involved in Lipid Metabolism

**Figure 5:**
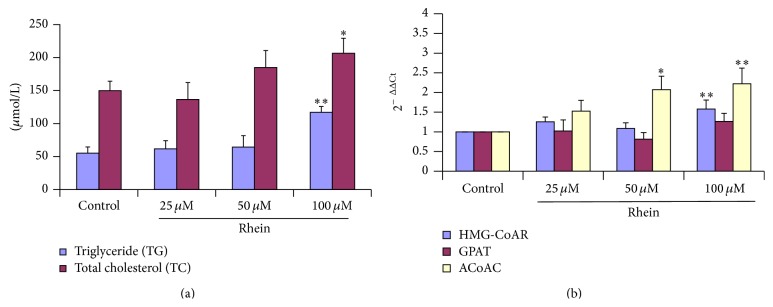
(a) Triglyceride (TG) and total cholesterol (TC) studies. Rhein induced* in vitro* triglyceride and total cholesterol upregulation in a dose-dependent manner. HL-7702 cells were incubated with different concentration of rhein for 12 h and then processed for TG and TC colorimetric assay. Analysis of TG and TC elevation was made relative to untreated control cells (100%). Data are expressed as mean ± SD from three independent experiments. (b) Quantitative analysis of HGM-CoAR, ACoAC, and GPAT mRNA expression levels in HL-7702 cells exposed to different doses of Rhein. GAPDH was used as internal positive control standard. The relative expression of target genes was calculated using 2^−ΔΔCt^ method. Data are expressed as mean ± SD from three different experiments. (^*∗*^ *P* < 0.05 versus control and ^*∗∗*^ *P* < 0.01 versus control).

Treatment of primary human hepatic HL-7702 cells with 100 *μ*M rhein for 12 hours revealed a significant biosynthesis of triglycerides (TG) and total cholesterol (TC) (Figure 5(a)). An increase in their serum levels can reflect active hepatocellular damage, which is of value as markers of chronic exposure to rhein as lipid accumulation in the liver is the major hallmark of nonalcoholic fatty liver disease (NAFLD).

HGM-CoAR, GPAT, and ACoAC are three key enzymes involved in lipid regulation metabolism. Their activity is considerably induced in liver damage. After 12 h of rhein exposure, the transcripts analysis showed significant statistical upregulation of these three enzymes in HL-7702 cells (Figure 5(b)). Thus, these results indicated that toxic effects of rhein could alter lipid regulation metabolism.

### 3.5. Measurement of Mitochondrial Membrane Potential (Δ*φ*_mit_)

**Figure 6:**
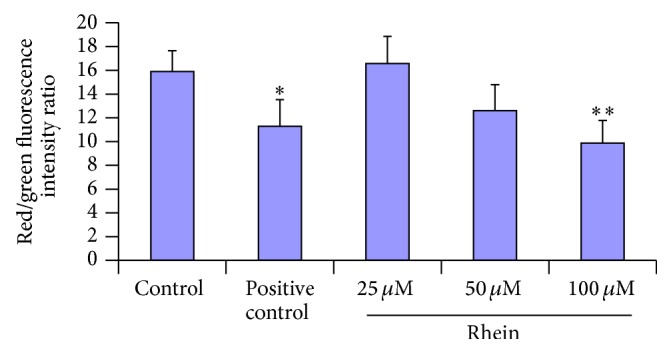
Mitochondrial membrane potential (Δ*φ*_mit_) detection by measurement of JC-1 fluorescence in HL-7702 cells in a 96-well plate format. The fluorescent intensity for both J-aggregates and monomeric forms of JC-1 was measured with a 96-well plate reader (J-aggregates: excitation/emission = 525/590 nm; JC-1 monomers: excitation/emission = 490/530 nm). The JC-1 red/green fluorescence intensity ratio was statistically significant in 100 *μ*M rhein treated cells. We also observed statistical significance in positive control group (CCCP). Data are expressed as mean ± SD from three independent experiments (^*∗*^ *P* < 0.05; ^*∗∗*^ *P* < 0.01; versus control group).

Given that Δ*φ*_mit_ is abrogated during apoptosis, we evaluated Δ*φ*_mit_ dissipation using the cationic lipophilic probe JC-1. The red/green ration in HL-7702 cells was significantly decreased by 100 *μ*M rhein treatment for 12 h (*P* < 0.01) or the 10 *μ*M CCCP treatment (*P* < 0.05), as the results are shown in Figure 6. CCCP was the positive control that could induce mitochondria membrane depolarization. The loss of Δ*φ*_mit_ was especially significantly elicited by exposure to rhein (50 *μ*M and 100 *μ*M) with comparison to control.

### 3.6. Reactive Oxygen Species (ROS) and Lipid Peroxidation

**Figure 7:**
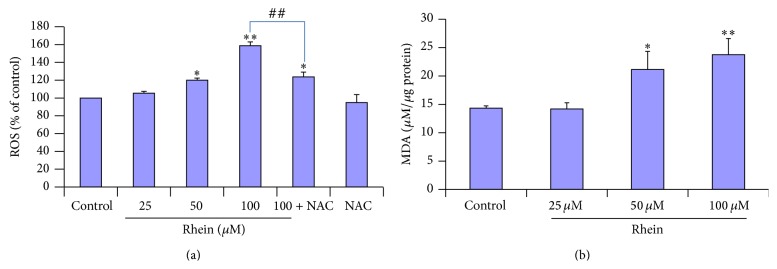
Rhein-induced oxidative stress in HL-7702 cells. (a) The cells were pretreated without or with 5 mM NAC for 1 h and then treated with rhein for 12 h. The generation of ROS was measured fluorometrically by using the fluorescent dye DFC-DA. The intracellular ROS production significantly increased after 50 *μ*M rhein and 100 *μ*M rhein. NAC was significantly effective in preventing ROS production in response to rhein (100 *μ*M). Data are expressed as mean ± SD from three independent experiments. ^*∗*^ *P* < 0.05, ^*∗∗*^ *P* < 0.01 versus control; ^##^ *P* < 0.01 compared to rhein (100 *μ*M). (b) Cells exposed for 12 h to rhein were used to analyze colorimetrically the oxidative deterioration of lipids to certain extend. The intracellular lipid peroxidation activity significantly increased after 50 *μ*M and 100 *μ*M rhein exposure. Data were expressed as mean ± SD from three different experiments, ^*∗*^ *P* < 0.05, ^*∗∗*^ *P* < 0.01, significantly different from control values.

Mitochondrion is considered to be a major site of ROS production that can be involved in cell death and ROS burst in mitochondrion may cause mitochondrial dysfunction as accumulating evidences support [48]. To determine whether this event occurs in rhein-induced apoptosis, we examined the intracellular production of ROS by fluorescence spectrophotometry, using DCFH-DA assay. HL-7702 cells were exposed to rhein at 25 *μ*M, 50 *μ*M, and 100 *μ*M for 12 h. The intracellular ROS production results are shown in Figure 7(a). The increase of intracellular ROS production was significantly elicited by exposure to 50 *μ*M rhein and 100 *μ*M rhein, compared to control. To further confirm that ROS acted as initiators in rhein-induced HL-7702 cells apoptosis, the cells were preincubated with 5 mM NAC prior to 100 *μ*M rhein for 12 h. As expected, the ROS scavenger significantly decreased the level of ROS to 121.67 ± 2.23%, ^##^ *P* < 0.01 (Figure 7(a)).

To elucidate the effects of rhein on oxidative damage in the HL-7702 cells to a certain extent, the content of MDA was determined. MDA, a secondary product of lipid peroxidation, is frequently used as indictor of tissue damage [49]. The radical formation resulting in lipid peroxidation is measured as MDA. Lipid peroxidation content (MDA) in cells was significantly increased (21.16 and 23.74, resp., ^*∗*^ *P* < 0.05 and ^*∗∗*^ *P* < 0.01) in response to rhein 50 *μ*M and 100 *μ*M for 12 h, compared to the corresponding control (Figure 7(b)).

### 3.7. Effect of Rhein Treatment on the Antioxidant Enzyme

**Figure 8:**
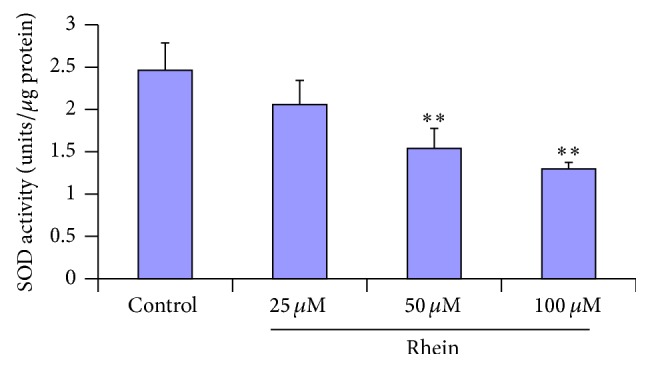
Total superoxide dismutase (SOD) was assessed in HL-7702 cells exposed to rhein for 12 h. Cells exposed for 12 h to rhein were used to analyze colorimetrically the SOD activity. The intracellular SOD activity significantly decreased after 50 *μ*M and 100 *μ*M rhein exposure. Data were expressed as mean ± SD from three different experiments, ^*∗∗*^ *P* < 0.01, significantly different from control values.

Increased activities of many antioxidant enzymes in cell reflect a defense on oxidative stress induced by drugs or environmental stress. Thus maintaining a high antioxidant capacity to scavenge the toxic ROS is critical for the cell life [50]. SOD, a scavenger of superoxide, is the most important protective enzyme that provides the first line of enzymatic antioxidant defense against oxidative stress in the liver [51]. The activities of liver antioxidant SOD (1.53 and 1.29, resp., ^*∗∗*^ *P* < 0.01) were significantly decreased in a dose-dependent manner in response to rhein treatment as compared to the corresponding control (Figure 8). These results suggested that rhein induces oxidative damage in HL-7702 cells.

### 3.8. Rhein Activated Caspases in HL-7702 Cells

**Figure 9:**
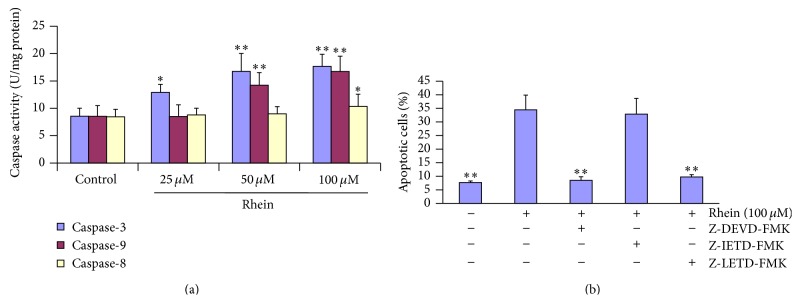
(a) Rhein-induced differential activation of caspase-3, caspase-8, and caspase-9 in HL-7702 cells following treatment with rhein (25 *μ*M, 50 *μ*M, and 100 *μ*M) for 12 h. Data are expressed as mean ± SD from three independent experiments (^*∗*^ *P* < 0.05, ^*∗∗*^ *P* < 0.01 versus control group). (b) Effect of caspase inhibitors on apoptosis in HL-7702 cells following treatment with rhein (100 *μ*M) for 12 h. Data are expressed as mean ± SD from three independent experiments, ^*∗∗*^ *P* < 0.01 compared to rhein (100 *μ*M) treated cells.

Despite varying conditions that can lead to apoptosis, caspase activation remains a universal event because the caspase family of cysteine proteases plays an important role in apoptosis and has been recognized as hallmarks of apoptosis [52]. To determine whether caspases are attributed to rhein-induced apoptosis in HL-7702 cells, caspase-3, -8, and -9 activities were detected. Results showed that rhein significantly increased caspase-3, -8, and -9 activities in dose-dependent manner, with maximum activities at 100 *μ*M (Figure 9(a)). However, the threshold concentrations for caspase-3, -8, and -9 activations were 25 *μ*M, 100 *μ*M, and 50 *μ*M, respectively.

To confirm that caspase activation is a key step in rhein-induced apoptosis, HL-7702 cells were pretreated with 25 *μ*M of Z-DEVD-FMK (caspase-3 inhibitor), Z-IETD-FMK (caspase-8 inhibitor), and Z-LETD-FMK (caspase-9 inhibitor) for 1 h and then subsequently exposed to 100 *μ*M rhein for 12 h. As shown in Figure 9(b), caspase-9 inhibitor and caspase-3 inhibitor significantly inhibited the antiproliferative activity of rhein. Rhein significantly triggered caspase protease activity in HL-7702 cells, and pretreating cells with inhibitors of caspase-9 and caspase-3, respectively, could lead to significant abolishing of rhein-induced caspase activity (*P* < 0.01), whereas pretreatment with caspase-8 inhibitor could not prevent the apoptosis induced by rhein in HL-7702 cells (*P* < 0.01). Therefore, based on these significant evidences, rhein-induced apoptosis in HL-7702 cells could be mediated through mitochondria-dependent pathway.

### 3.9. Relative mRNA Levels in Rhein-Induced Apoptotic HL-7702 Cells

**Figure 10:**
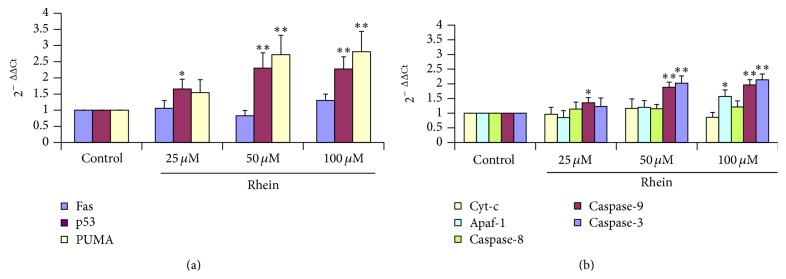
Quantitative analysis of (a) p53, PUMA and Fas and (b) Cyt-c, Apaf-1, Casp-8, Casp-9, and Casp-3 mRNA expression levels in HL-7702 cells exposed to different doses of rhein. GAPDH was used as an internal positive control standard. The relative expression of target genes was calculated using 2^−ΔΔCt^ method. Data were expressed as mean ± SD from three different experiments, ^*∗*^ *P* < 0.05, ^*∗∗*^ *P* < 0.01, significantly different from control values.

The induction of rhein has been shown to be significantly involved in apoptosis by caspase-dependent pathway. Apoptosis is induced by p53 via transcription-dependent and transcription-independent processes. Apoptosis can be induced by either the intrinsic mitochondrial pathway or the extrinsic cell death receptor pathway. To gain a broader understanding of molecular mechanisms for rhein's biological effects in primary human liver HL-7702 cells, by trying to elucidate the upstream molecular events leading to the activation of caspase-8 and caspase-9 upon rhein stimulation, gene expression analysis was carried out. We determined whether regulation of p53, PUMA, Fas, Cyt-c, Apaf-1, and Casp-8, Casp-9, and Casp-3 was mediated via modulating the expression of their respective mRNA through quantitative real-time PCR. Their specific products generated by qRT-PCR were normalized with respect to GAPDH. Results are summarized in Figure 10. Rhein at the three different concentrations (25 *μ*M, 50 *μ*M, and 100 *μ*M after 12 h exposure) significantly increased the expression of p53 mRNA and PUMA mRNA (Figure 10(a), *P* < 0.01), whereas it also significantly increased the expression of Apaf-1, Casp-8, -9, and -3 mRNA. In the same time, rhein did not significantly change the expression of Fas mRNA and Cyt-c mRNA (Figure 10(b), *P* < 0.01). Consequently, quantitative real-time RT-PCR analysis significantly revealed and strengthened the evidences that rhein-induced apoptosis in HL-7702 cells is principally mediated through intrinsic (mitochondria) pathway.

### 3.10. Effect of Rhein on Expressions of Apoptosis-Related Proteins

**Figure 11:**
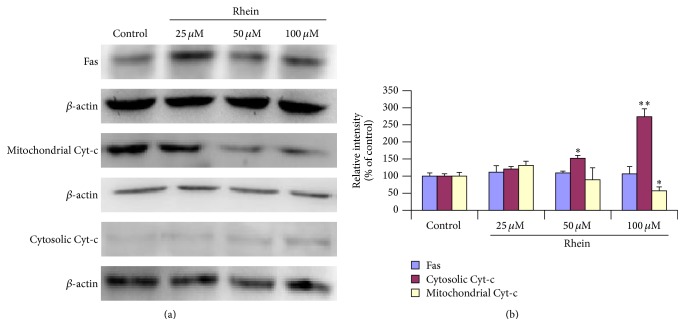
Western blot analysis of Fas and mitochondrial/cytosolic Cytochrome-c protein expression levels in HL-7702 cells exposed to different doses of rhein. (a) Representative results of western blotting analysis. *β*-actin served as a loading control. (b) The quantification of the immunoblots was analyzed by densitometric scanning. Band densities were digitized and relative band intensities of target proteins were normalized against the *β*-actin levels. Data were expressed as mean ± SD from three different experiments, ^*∗*^ *P* < 0.05, ^*∗∗*^ *P* < 0.01, significantly different from control values.

To further investigate the mechanism of rhein-induced apoptosis in HL-7702 cells, western blot analysis was performed to examine the protein expression levels of Fas, mitochondrial Cyt-c, and Cytosolic Cyt-c in the treated cells. Immunoblots are shown in Figure 11(a). As indicated in Figure 11(b) levels of cytosolic Cyt-c protein were significantly increased in a concentration-dependent manner after treatment with rhein. Compared to the control, the increase was significant with 50 *μ*M and 100 *μ*M for 12 h (*P* < 0.01). In contrast the levels of mitochondrial Cyt-c protein were significantly decreased after incubation with rhein at 100 *μ*M over a period of 12 h (*P* < 0.05). Meanwhile the levels of Fas protein did not show any significant changes. The leakage of Cytochrome-c from mitochondria into the cytosol and the no expression of Fas related protein (Figure 11) significantly strengthened and corroborated the hypothesis that rhein-induced apoptosis in HL-7702 cells is mediated through mitochondria pathway.

## 4. Discussion

Herbal medicines have been increasingly used worldwide as they often regarded by the public as harmless remedies for a variety of medical ailments [53]. However, recently researchers had paid special attention to herbal hepatotoxicity in order to insure herbal medicines safe dosage and toxicity profile [12]. Several clinical cases of* Polygonum multiflorum*-induced hepatotoxicity have been reported worldwide. In order to understand the toxicological effect and mechanism of* Polygonum multiflorum* and to investigate a potential clinical significance for ensuring the safety of administration herbal medicines, we conducted series of experimental procedures of rhein (one of the main bioactives of* Polygonum multiflorum*) on primary human noncarcinogenic HL-7702 cells, a widely used model cell line for toxicity model. The cytotoxicity studies conducted in the primary human hepatic cells after rhein exposure at different concentration for 24 h and 48 h suggested that rhein could induce inhibiting cell viability in primary human hepatic cells in dose- and time-dependent manner (Figure 2). IC_50_ values were calculated as 131.79 *μ*M and 18.45 *μ*M, at 24 h and 48 h, respectively. A 48 h acute toxicity study done by Bironaite and Ollinger [54] has found that LD_50_ of rhein in primary cultures of rat hepatocytes was 20 *μ*M. Toxicity of rhein was further tested by Mahbub et al. [55] in their 24 h acute toxicity study, where human cancer cells were treated with rhein at various doses (2–500 *μ*M). It was found that the IC_50_ of rhein was equal or more than 135 *μ*M, suggesting that the effectiveness of this polyphenol varies depending on the leukemia cell lineage (lymphoid versus myeloid) and in some cases within the cell lines from the same lineage [55]. Our study showed that after treating the human primary liver cells with rhein at various concentrations (0.12–370 *μ*M); the IC_50_ values were calculated as 131.79 *μ*M and 18.45 *μ*M at 24 h and 48 h, respectively. These values were relatively lower than the ones from the two previous studies mentioned [54, 55], suggesting a comparative corroboration with the concentrations of rhein used in our study.

Our data from the LDH release experiments (Figure 3(a)) showed that, with a threshold concentration of 50 *μ*M, rhein ravaged the integrity of the cytomembrane, causing apparent LDH leakage. Characteristic morphological features or alterations such as cell shrinkage, chromatin condensation, and formation of apoptotic bodies are associated with apoptotic cells. To explore the apoptosis-inducing effect of rhein, we carried out an observation of the cell outline and nucleus using a light and fluorescence microscopy and came to the significant results that HL-7702 cells treated with rhein exhibited the typical apoptotic morphological features (Figure 3(b)). ALT and AST are cytosolic enzymes mainly found in the liver. Their levels are valuable aid primarily in the diagnosis of liver disease; it can be used in combination with other enzymes to monitor the courses of various liver disorders as they are also biological catalysts. Their overconcentration in hepatocytes is quite a histopathological indicator of hepatocellular injury [56]. Figure 3(c) suggested a hepatocellular injury, thus asserting the fact that rhein could induce* in vitro* toxicity in primary human hepatic cells, by destroying the cytomembrane. These aforementioned apoptotic morphological features were more evident as we carried out a flow cytometry assay. The results of the experiment have indicated that rhein has dose-dependent toxic effects on HL-7702 cells and were consistent with the previous observations of changes in cellular ultrastructure, confirming 50 *μ*M as an apparent threshold concentration of rhein-inducing cytotoxicity in HL-7702 cells (Figure 4(b)).

The liver plays a key role in regulation of whole body lipid; thus lipid deposition in the liver is associated with metabolic disorders including fatty liver disease, type II diabetes, and hepatocellular cancer [57]. In order to broaden our understanding to the investigation in the mechanisms involved in rhein-induced apoptosis in the primary human hepatic cells, we investigated the behavior of some lipid and relevant genes as lipid metabolism plays important role in the life activities. Isolated hepatocytes undergo lipoapoptosis, a feature of hepatic lipotoxicity, on treatment with saturated free fatty acids [58]. Lipoapoptosis occurring due to an excess of saturated free fatty acids is a key pathogenic event in the initiation of nonalcoholic fatty liver disease. Although cholesterol plays a vital role in regulating physical properties of membranes [59], its accumulation in cells is toxic and causes fatal diseases such as Niemann Pick type C diseases, which is a fatal neurodegenerative disease and the second most common cause of neonatal cholestasis, characterized by lysosomal storage of cholesterol and glycosphingolipids [60]. Moreover elevated levels of cholesterol and triglycerides (Figure 5(a)) have been linked to liver diseases, as triglyceride deposition within the hepatocyte is the hallmark of both alcoholic and nonalcoholic fatty liver diseases [61]. HMG-CoAR transcripts are highly enriched in liver cells (hepatocytes), where cholesterol is converted into bile salts and where lipoproteins involved in transporting cholesterol are synthesized and exported. GPAT is involved in the first step in glycerolipid synthesis and is localized in both the endoplasmic reticulum and mitochondria. Moreover ACoAC catalyzes the formation of malonyl-CoA which, in turn, is utilized by the fatty acid synthetase complex for the de* novo* synthesis of fatty acids. HMG-CoAR, GPAT, and ACoAC are commonly overexpressed or overactivated in diseases states associated with fatty liver or liver damaged [62–65]. In the present study, the upregulation of genes involved in lipid synthesis in rhein treated HL-7702 hepatic cells such as HMG-CoAR, ACoAC, and GPAT (Figure 5(b)) could lead to increased glycerolipid and cholesterol biosynthesis. Experimental studies have shown higher lipid levels after exposure to* Polygonum multiflorum* impaired normal cell signaling and causing cellular dysfunction [66, 67]. Based on the aforementioned results, the findings suggested that lipid overload and fatty degeneration could be involved in rhein-induced cell death.

Mitochondria, as dynamic organelles, have a crucial role in maintaining both cellular bioenergetics and regulating signaling pathways to meet the high energy demands in the cells. Thus, any alterations to the mitochondrial homeostasis will lead to loss of integrity or damage resulting to apoptosis [68]. While mitochondria consume oxygen and substrates to generate ATP, they produce reactive oxygen species in the process. In mitochondria, Cytochrome-c is required as an electron carrier in oxidative phosphorylation and shuttles electrons from one complex (Complex III) to another (Complex IV); however the electron transport between these two complexes generates a proton gradient across the inner mitochondrial membrane, which maintains Δ*φ*_mit_ [69]. Cytochrome-c release from mitochondria is a key step of apoptosis [70]. The mitochondrial dysfunction features including loss of mitochondrial membrane potential (Δ*φ*_mit_) and leakage of Cytochrome-c from the mitochondrion into the cytosol were also investigated in our study. In this study, the significant loss of Δ*φ*_mit_ (Figure 6) and the translocation of Cytochrome-c (Figures 11(a) and 11(b)) were significantly observed in rhein-treated HL-7702 cells. Moreover, excessively production of ROS (Figure 7(a)) may alter the mitochondria membrane [48], causing a disruption of Δ*φ*_mit_ and the release of Cytochrome-c [71], that in turn triggers mitochondrial membrane permeability and apoptosis. By immunofluorescence staining, we found the release of Cytochrome-c from mitochondria (Figure 11(a)). These findings suggested that the cascade reactions of ROS production, lipid peroxidation, loss of Δ*φ*_mit_, and release of Cytochrome-c from mitochondria may play an involved role in rhein-induced apoptosis in HL-7702 cells in a dose-dependent manner.

Oxidative stress and liver injury are strongly associated. Oxidative stress in the liver can be triggered during different conditions and by specific etiologies, including hepatotoxins such as rhein. Oxidative stress is a state of imbalance between the production of reactive oxygen species (ROS) and the cellular antioxidant defense neutralizing the reactive intermediates and triggering damage. At a physiological level, mitochondria are primary source of ROS. Moreover ROS is involved in regulation of the intracellular signaling pathways as “redox messenger,” whereas excessive production of ROS can lead to lipid peroxidation, mitochondrial oxidative stress, and DNA damage, inducing oxidative modification of cellular macromolecules, inhibit protein function, and promote apoptotic cell death [48, 72–74]. Meanwhile, experiments showed that ROS act upstream of mitochondrial membrane depolarization, Cytochrome-c release, execution of caspase activation, and nuclear fragmentation [75]. In our current study, we observed the significant excess production of ROS in HL-7702 cells especially with rhein at 50 *μ*M and 100 *μ*M (Figure 7(a)). Pretreatment with ROS scavenger NAC could impressively reverse the action triggered by 100 *μ*M rhein (Figure 7(a)). In a comparative study of three anthraquinones (rhein, danthron, and chrysophanol) done on primary cultures of rat hepatocytes [36] only rhein at 50 *μ*M was found to be the most effective in producing free radicals and was the only tested compound that could induce apoptosis. The* in vitro* doses of rhein used in our study and the pharmacological outcome (apoptosis) observed here in HL-7702 cells were significantly in accordance with previous studies [36]. Rhein, which contains one carboxyl group, is suitable for one-electron-reducing enzymes and an effective redox cycler, which leads to the production of oxygen-derived free radicals that eventually induced apoptotic cell death [36]. Lipid peroxidation is one of the main manifestations of oxidative damage initiated by ROS and it has been linked to the altered membrane structure and enzyme inactivation and excessive damage, leading to cell death [76]. In our study (as shown in Figure 7(b)), rhein may facilitate these deleterious effects by promoting the lipid peroxidation process, thus increasing the formation of MDA. The amount of MDA was significantly high in cells treated with rhein at 50 *μ*M (^*∗*^ *P* < 0.05) and 100 *μ*M (^*∗∗*^ *P* < 0.01). Cells maintain a variety of defenses in response to oxidative stress through the induction of antioxidant enzymes with SOD being one of the most important endogenous enzymatic antioxidants. In our study, SOD activity was significantly decreased by treatment with rhein (from 50 *μ*M) at 12 h (Figure 8). Previous studies done on HL-7702 cell suggested that oxidative stress is associated with apoptosis in this same cell line [77–79]. Furthermore, a study of rhein's metabolism done in primary cultures rat hepatocytes caused production of oxygen-derived free radicals by redox cycling, initiation of lipid peroxidation which eventually led to cell death [54]. The activities of ROS, superoxide dismutase, and lipid peroxidation serve as reliable indicators of oxidative damage. In our study, these results indicated that treatment with rhein induced increase in ROS and MDA and loss in activity of SOD, which resulted in oxidative stress and the concentration-dependent increases of apoptotic cells. NAC treatment was able to ameliorate the oxidative stress. Based on the evidences reported above, there is a reason to speculate that oxidative stress could be involved in rhein-induced apoptosis in HL-7702 cells and these results are consistent with the ROS-mediated toxicity of medicinal herbs in the same cell line.

The p53 network mediates cellular responses to diverse forms of stress (e.g., DNA damage, oncogene activation, and hypoxia). Functioning primarily as a transcription factor, p53 regulates the expression of genes involved in cell cycle arrest, DNA repair, senescence, and apoptosis [80]. It can regulate the intrinsic mitochondrial-mediated apoptotic pathway and the extrinsic apoptotic pathway. In the intrinsic mitochondrial pathway, p53 induces transcription of several genes such as PUMA (p53 upregulated modulator of apoptosis) which has an expression pattern consistent with a causative role in p53-dependent apoptosis [81]. Upon p53 activation, resulting in functional PUMA mRNA accumulation confirmed the results of our RT-qPCR analyses (Figure 10(a)). These observations in correlation with the results of other* in vitro* studies [80] suggested that PUMA a major effector of p53-mediated cell death may play an important role in* in vitro* regulator of apoptosis when it overexpressed. Moreover the results of the overexpression of p53 mRNA and PUMA mRNA (Figure 10(a)) taken together corroborated the findings expressed by Tsai and Barton [82], suggesting apparently that the branch of apoptosis put in evidence here was the intrinsic mitochondrial pathway. Following cellular insults that cause DNA damage, such as ionizing radiation (IR), ultraviolet (UV) radiation, and oxidative stress, may lead to p53 upregulation which afterward this cascade of events will eventually lead to apoptosis [77].

In the intrinsic mitochondrial pathway, p53 induces PUMA and as a result we assist to the mitochondrial membrane depolarization causing the release of Cytochrome-c. After release from mitochondria, Cytochrome-c binds to Apaf-1, which additionally is a direct target of p53-regulated activation of transcription. Afterwards, there is execution of caspase activation. The caspase family of cysteine proteases plays an important role in apoptosis; therefore the caspase activation is considered as an apoptotic marker. Moreover, the apoptosis can be either intrinsic pathway, involving mitochondrial injury and caspase-9 activation, or extrinsic pathway due to Fas/FasL receptor-mediated caspase-8 activation, both consequently leading to the activation of caspase-3 (known as executor of cell death). In our study, to our surprise caspase-3, caspase-8, and caspase-9 were all activated in rhein-treated HL-7702 cells by activation analysis (Figure 9(a)). Additionally, caspase-9 or caspase-3 inhibitor could stop rhein-inducing apoptotic effect (Figure 9(b)), suggesting that rhein is able to induce apoptosis in HL-7702 cells through mitochondria-mediated pathway. The activation of caspase-9 resulted from the leakage of Cytochrome-c, and then the activated caspase-9 could induce activation of caspase-3. The RT-qPCR analysis showed a significant upregulation of Apaf-1 and Casp-9 and -3 transcripts (Figure 10(b)), confirming the intrinsic apoptotic pathway. However, to our surprise, even though the expression of Fas from the RT-qPCR (Figure 10(a)) and the western blots analysis (Figure 11(b)) did not reveal any significant changes, but caspase-8, belonging to the death receptor apoptotic pathway, was significantly activated (Figure 9(a)). This could be explained as scientific evidences suggested that caspase-8 can be activated in the early stage as an initiator caspase by activated caspase-3 [83, 84]. The activation of Apaf-1 resulted from the leakage of Cytochrome-c; triggering the upregulation of caspace-9 and then the activated caspase-9 could induce activation of caspase-3 (Figure 10(b)). Therefore, the present results suggested that the activation of caspase-8 might be induced by the activated caspase-3 and strengthening the confirmation that based on the present aforementioned results, the mechanism of rhein-induced apoptosis in primary human liver HL-7702 cells is through intrinsic mitochondria- mediated pathway.

Pharmacokinetics analyses have been conducted to investigate the rational clinical dose of rhein from herbal formulas. Several pharmacokinetics studies done on rats after oral administration of rhein at 70 mg·kg^−1^ [85], 11.9 mg·kg^−1^ [86], and 70 mg·kg^−1^ [87] have led to analyze rhein's pharmacokinetics parameters to a certain extent, suggesting that rhein has a rapid absorption and a slow elimination. For calculations of determining the starting dose in humans as extrapolated from animals, scientists have used normalization of body surface area (BSA) method [88] and the conversation of animals dose to human equivalent doses based on BSA [89], respectively, shown in (1) and Table 2 as follows:

 Formula for dose translation based on BSA [83]
  (1) $$\begin{array}{c} \text{HED }\left(\;\text{mg/kg}\;\right)=\text{Animal dose }\left(\;\text{mg/kg}\;\right)\frac{\text{Animal }K_{m}}{\text{Human }K_{m}}, \end{array}$$

where HED is human equivalent dose and BSA is body surface area. Clinical studies done in human by Tan et al. [90], Zhu et al. [91], Jiang et al. [92], and Hao et al. [87] demonstrated that linear pharmacokinetics for rhein in Chinese healthy patients after a single oral administration is in the range of 50–200 mg (equivalent to 1.7 and 6.7 times of the upper dose of human stipulated in China Pharmacopoeia (0.5 g·kg^−1^)) [93] and enlightened that the pharmacokinetics parameters of rhein as a single compound are significantly different from those of rhein as a compound in a herbal plant or formula. Further, the clearance scaling of* in vivo-in vitro* in the same species approach has been shown to be successful for* in vivo-in vitro* data of rhein, using a physiological based pharmacokinetic (PBPK) model [87]. Based on the results of these toxicity studies, there is a clear understanding of the pharmacokinetic behavior of rhein as a single drug and as a component of herbal formulae, leading us to further investigating the extrapolated dosed in various clinical study models.

## 5. Conclusion

To date, the hepatotoxicity reported from herbal medicines or related herbal bioactive component still remains an important issue to address for drug safety in clinical application. In this present study, we demonstrated that rhein is able to decrease primary human hepatic HL-7702 cells viability. Rhein exerted toxicological effects in HL-7702 cells that could be done via mitochondria-mediated pathway in a dose-dependent manner. The apoptosis induced by rhein (50 *μ*M and 100 *μ*M for 12 h) in HL-7702 cells is associated with several morphological changes and biochemical signals such as the following: (i) oxygen radicals that can affect the permeability and potential of the inner mitochondrial membrane, (ii) leakage of Lactate dehydrogenase, (iii) overproduction of ROS, lipid peroxidation, loss in activities of SOD, (iv) dysregulation of enzymes involved in lipid metabolism, (v) loss of Δ*φ*_mit_, leaking Cytochrome-c from mitochondria into cytosol, and (vi) subsequently enhancing PUMA, Apaf-1, and caspase-9 and -3 activities. Based on all evidences reported above, these findings provide a mechanistic explanation for the hepatotoxicity of rhein in drug-induced oxidative liver injury from herbal medicine. To our knowledge, this is the first report of rhein-induced apoptosis in primary human hepatic HL-7702 cells. The findings of our study are conducive to further conduct both* in vitro* and* in vivo* pharmacokinetics studies with primary human HL-7702 liver cells as hepatic support system in order to investigate and ensure the clinical administration safety of* Polygonum multiflorum* or associated herbs containing rhein and other main phytochemical compounds and moreover to investigate various signaling involved in herbal hepatotoxicity.

## Figures and Tables

**Table 1:** Sequences of the primers used in real-time PCR.

Gene description	Primer	Sequence (5′→3′)	Length (bp)
GAPDH	GAPDH-F	CAGGAGGCATTGCTGATGAT	20
	GAPDH-R	GAAGGCTGGGGCTCATTT	18

HGM-CoAR	HGM-CoAR-F	AGCCTGAATAGCCCGACAG	19
	HGM-CoAR-R	CATCCTCCACAAGACAATGC	20

GPAT	GPAT-F	TGAACAACTGGGCAAACCTAA	21
	GPAT-R	AAATCCACTCGGACACAACC	20

ACoAC	ACoAC-F	CTCTTGACCCTGGCTGTGTA	20
	ACoAC-R	GATGGAGTTTCTCGCCTCTG	20

p53	p53-F	GCCATCTACAAGCAGTCACAG	21
	p53-R	ATTTCCTTCCACTCGGATAAGA	22

PUMA	PUMA-F	GAAGAGCAAATGAGCCAAAC	20
	PUMA-R	CAGAGCACAGGATTCACAGTCT	22

Fas	Fas-F	ACACTCACCAGCAACACCAAGT	22
	Fas-R	CCTTTCTCTTCACCCAAACAAT	22

Cyt-c	Cyt-c-F	TACTCTTACACAGCCGCCAATA	22
	Cyt-c-R	AGTCTGCCCTTTCTTCCTTCTT	22

Apaf-1	Apaf-1-F	GTGAAGTGTTGTTCGTGGTCTG	22
	Apaf-1-R	CGTGTGGATTTCTCCCAATAG	21

Casp-8	Casp-8-F	ATGTTGGAGGAAAGCAATCTGT	22
	Casp-8-R	CCTGCCTGGTGTCTGAAGTT	20

Casp-9	Casp-9-F	ACTAACAGGCAAGCAGCAAAGT	22
	Casp-9-R	ACATCACCAAATCCTCCAGAAC	22

Casp-3	Casp-3-F	AGCAATAAATGAATGGGCTGAG	22
	Casp-3-R	GTATGGAGAAATGGGCTGTAGG	22

**Table 2:** Conversion of animal doses to HED based on BSA.

Species	Weight (kg)	BSA (m^2^)	*K*_*m*_ factor
Human			
Adult	60	1.6	37
Child	20	0.8	25
Baboon	12	0.6	20
Dog	10	0.5	20
Monkey	3	0.24	12
Rabbit	1.8	0.15	12
Guinea pig	0.4	0.05	8
Rat	0.15	0.025	6
Hamster	0.08	0.02	5
Mouse	0.02	0.007	3

Values based on data from FDA Draft Guidelines [84].

HED: human equivalent dose, BSA: body surface area.

To convert dose in mg/kg to dose in mg/m^2^, multiply by  *K*_*m*_ value.

## Abbreviations

PMT: * Polygonum multiflorum* Thunb.
HL-7702 cells: Primary human liver cells
DILI: Drug-induced liver injury
PCD: Programmed cell death
PS: Phosphatidylserine
UGT: Uridine diphosphate glucuronosyltransferase
ALF: Acute liver failure
ALT: Alanine aminotransferase
AST: Aspartate aminotransferase
TG: Triglyceride
TC: Total cholesterol
PMSF: Phenylmethylsulfonyl fluoride
NAC: N-Acetyl-L-cysteine
MDA: Lipid peroxidation
SOD: Total superoxide dismutase
HMG-CoAR: Hydroxymethylglutaryl coenzyme A reductase
ACoAC: Acetyl-CoA carboxylase
G3P: Glycerol-3-phosphate
LPA: Lysophosphatidic acid
GPAT: Glycerol-3-phosphate acyltransferase
Δ*φ*_mit_: Mitochondrial membrane potential
CCCP: Carbonyl cyanide m-chlorophenylhydrazone
DCFH-DA: Dichloro-dihydro-fluorescein diacetate
ROS: Reactive oxygen species
GAPDH: Glyceraldehyde-3-phosphate dehydrogenase.
